# Oxides for Rectenna Technology

**DOI:** 10.3390/ma14185218

**Published:** 2021-09-10

**Authors:** Ivona Z. Mitrovic, Saeed Almalki, Serdar B. Tekin, Naser Sedghi, Paul R. Chalker, Stephen Hall

**Affiliations:** 1 Department of Electrical Engineering and Electronics, University of Liverpool, Liverpool L69 3GJ, UK; s.almalki2@liverpool.ac.uk (S.A.); nsed@liverpool.ac.uk (N.S.); s.hall@liverpool.ac.uk (S.H.); 2 School of Engineering, University of Liverpool, Liverpool L69 3GH, UK; pchalker@liverpool.ac.uk

**Keywords:** rectenna, MIM, diode, terahertz, infrared, oxide, energy harvesting

## Abstract

The quest to harvest untapped renewable infrared energy sources has led to significant research effort in design, fabrication and optimization of a self-biased rectenna that can operate without external bias voltage. At the heart of its design is the engineering of a high-frequency rectifier that can convert terahertz and infrared alternating current (AC) signals to usable direct current (DC). The Metal Insulator Metal (MIM) diode has been considered as one of the ideal candidates for the rectenna system. Its unparalleled ability to have a high response time is due to the fast, femtosecond tunneling process that governs current transport. This paper presents an overview of single, double and triple insulator MIM diodes that have been fabricated so far, in particular focusing on reviewing key figures of merit, such as zero-bias responsivity (*β*_0_), zero-bias dynamic resistance (*R*_0_) and asymmetry. The two major oxide contenders for MI^n^M diodes have been NiO and Al_2_O_3_, in combination with HfO_2_, Ta_2_O_5_, Nb_2_O_5_, ZnO and TiO_2_. The latter oxide has also been used in combination with Co_3_O_4_ and TiO_x_. The most advanced rectennas based on MI^2^M diodes have shown that optimal (*β*_0_ and *R*_0_) can be achieved by carefully tailoring fabrication processes to control oxide stoichiometry and thicknesses to sub-nanometer accuracy.

## 1. Introduction

Renewable energy sources are a key element in the drive towards zero-carbon economy targets worldwide in the decades to come. There has been unprecedented research activity towards sustainable energy materials and devices. Our earth receives ~1000 W/m^2^ at sea level during clear weather conditions. The major component of this solar energy of over 50% is in the infrared region (IR). The mid-IR wavelength range from 1.5 to 20 μm is the most important since it is re-emitted from the earth’s surface as IR radiation from 8 to 14 μm with maximum emissivity at 10.6 µm (28.3 THz). It is this energy that remains untapped by current solar cell-based harvesting sources. Conventional photovoltaic (PV) renewable technology has been used to harvest only the energy from the visible spectrum (400 to 750 nm) of the sun. Furthermore, unlike the energy of solar panels being limited by daylight and climate conditions, the energy of infrared heat can be harvested day and night. The concept of a rectifying antenna (rectenna) is not new [1,2], and its efficient design and operation in the IR region has been intriguing scientists for several decades. The use of an antenna and rectifying diode has only been successfully demonstrated for energy collection at microwave and radio frequencies [3]. An important point is that contrary to the photovoltaic technology where the conversion efficiency is limited by the semiconductor band gap, rectennas can, theoretically, achieve 100% conversion efficiency [4,5,6]. As such, the rectenna technology sits at the frontiers of high-frequency electronic devices and nanophotonics applications, to name just a few: optical transceivers in communication systems [7], IR and optical detection [8,9], and biosensors [10].

**Figure 1:**
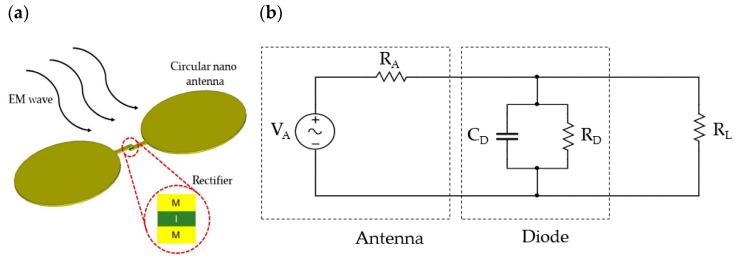
(**a**) Schematic of a rectenna device and (**b**) its equivalent circuit.

An optical rectenna consists of a receiving nano-antenna and a rectifying diode, as illustrated in Figure 1a. A metal-insulator-metal (MIM) diode has been considered as a prime contender for the rectenna system, and it is the main focus of review in this paper. The MIM diode can operate at terahertz (1 THz = 10^12^ Hz) frequencies due to the tunneling current mechanism having the electron transit time in femtoseconds (10^−15^ s). The rectification involves converting the oscillating charges (the alternating current (AC) electromagnetic signal), provided by the antenna arms flowing through the insulating thin film of a MIM rectifier, into a direct current (DC) signal. A simple equivalent circuit of a rectenna is shown in Figure 1b. It consists of an alternating voltage source (V_A_) with associated antenna resistance (R_A_) in series with the diode part comprising of the dynamic resistance (R_D_) and diode capacitance (C_D_) in parallel with the load resistance R_L_.

For efficient terahertz rectification, low diode capacitance and dynamic resistance are mandatory. The cut-off frequency (*f_c_*) can be calculated as:

(1) $$f_{c}=\frac{1}{2\pi R_{D}C_{D}}$$

**Figure 2:**
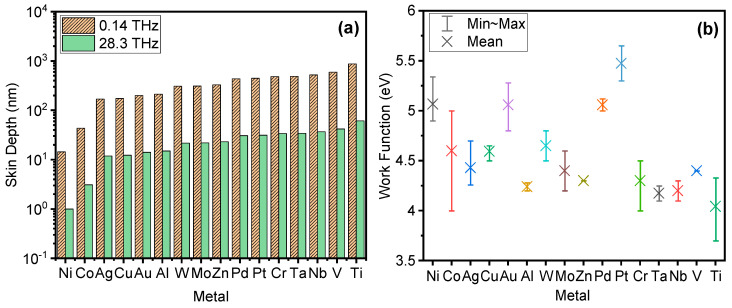
(**a**) Skin depth and (**b**) work function [11,12,13,14,15,16,17,18,19,20] for typical metals used in a rectenna.

Lowering the diode capacitance can be achieved by fabricating an MIM diode with minimal area or using a thick dielectric, since

(2) $$C_{D}=\frac{A\varepsilon_{r}\varepsilon_{o}}{d}$$

where d is the thickness of the insulator, $\varepsilon_{r}$—its relative permittivity, $\varepsilon_{o}$—permittivity of vacuum and *A* is the diode area. However, increasing the oxide thickness will increase the diode resistance, which leads to a reduction of the electron tunneling probability through a wider energy barrier. Moreover, the insulator thickness should be in the range of a few nanometers so that electron tunneling dominates other parasitic transport mechanisms. The selection of materials for both metals and insulators as well as the insulator thickness control the diode electrical characteristics. A selection of typical metals that have been used in MIM diode fabrication is shown in Figure 2 and Table 1 with values of work function as reported in the literature [11,12,13,14,15,16,17,18,19,20]. At terahertz frequencies, the metal conductivity decreases, hence the skin effect should be considered. It can be calculated using Equation (3) for each metal, where the thickness of metal should be about five times the skin depth value to maintain good conductivity in the metal at a high-frequency regime:

(3) $$\delta ={\left(\rho /\pi f\mu_{o}\mu_{r}\right)}^{1/2}\;$$

where δ is the skin depth, ρ is the resistivity of metal, $\mu_{r}$ is relative magnetic permeability of the metal, $\mu_{o}$ is the magnetic permeability of vacuum and *f* is the operating frequency. The skin depths for frequently used metals in rectennas that can operate at 0.14 and 28.3 THz are shown in Figure 2a and calculated using ρ and $\mu_{r}$ in [21,22,23,24,25,26]. It can be seen that Ni has the smallest skin depth, and some of the first successful demonstrations of bowtie rectenna designs were realized using Ni-NiO-Ni diodes [27,28]. In terms of work function, the lowest values have Co, Cr and Ti, although there is a large range of values reported, especially for the former two metals (Table 1, Figure 2b) [11,13,15,16]. Furthermore, conductive oxides (indium tin oxide (ITO) and SrRuO_3_ (SRO)) and nitrides (TiN and NbN, see References in [29]) as well as multicomponent amorphous metals (ZCAN (ZrCuAlNi) and TiAl_3_) [18,30,31] can also act as electrodes in MIM diodes.

**Figure 3:**
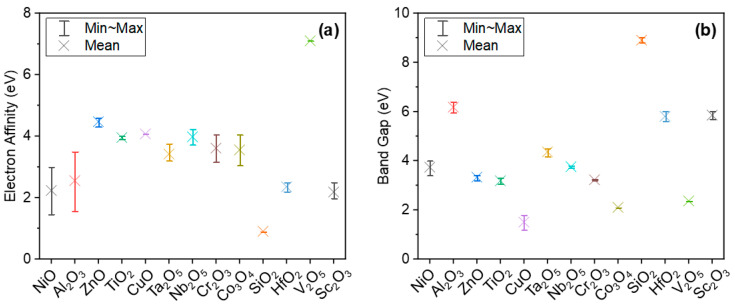
(**a**) Electron affinity and (**b**) band gap of typical oxides for MIM diodes. Full details are provided in Table 1.

Figure 3a,b present electron affinities [9,17,32,33,34,35,36,37,38,39,40,41,42,43,44] and band gap [33,35,36,38,43,45,46,47,48,49,50,51,52,53,54,55] of typical oxides that have been used in the design and fabrication of MIM diodes. Full details are listed in Table 1 for completeness. Note that the large variation of reported values for electron affinity for Al_2_O_3_, NiO, Co_3_O_4_ and Cr_2_O_3_ is likely to be related to the variations in fabrication conditions. Changes in work function and *χ* can be up to 1 eV for metals and semiconductors depending on the surface conditions. These changes are likely to be due to the formation of electronic dipoles at the surface, changing the minimum energy for an electron to leave the sample [45]. Furthermore, the reported measured values of oxide band gap (Table 1) are found to vary depending on the stoichiometry and structural properties (amorphous, crystalline, polycrystalline) of films fabricated by different deposition techniques. As can be seen from Table 1, the band gap of amorphous Al_2_O_3_ thin films prepared by atomic layer deposition (ALD) [38] or molecular beam epitaxy [47] is found to be ~6.4 eV, while non-stoichiometric AlO_x_ prepared by radio frequency (RF) sputtering exhibits a smaller band gap of 5.95 eV [45]. Another example is crystalline Sc_2_O_3_ films measured to have a band gap of 6.0 eV, while amorphous films have smaller values of ~5.7–5.8 eV [43]. Crystalline NiO was found to have a band gap of ~4.0 eV [46], while the most recent study shows a much smaller value of 3.4 eV for an RF-sputtered, 2 nm NiO film [45]. It is worth mentioning also that the variation of ±0.25 eV in reported band gap values in Table 1 could also be due to tolerances in using different characterization techniques to measure the band gap; that is, for Ta_2_O_5_, 4.2 eV from UV-vis (ultra violet-visible) absorbance spectra [35] in comparison to ~4.4 eV measured by reflection electron energy loss spectroscopy [38] or variable angle spectroscopic ellipsometry [51].

As stated in Equation (1), to achieve very small resistance × capacitance (RC) time constant and harvest IR energy, a trade-off between different physical parameters of diodes often limits practical implementation in rectennas. A number of significant issues need to be overcome, including (i) a precise manufacturing process with smooth metal electrodes and high-quality ultra-thin oxides, (ii) patterning of nano-scale devices as well as (iii) coupling efficiency and diode integration with the antenna. A rough metal surface affects oxide uniformity and hence the diode’s electrical characteristics. This eventually reduces the yield of functioning devices. Furthermore, even a small variation in oxide thickness largely affects the tunneling probability and the resulting current density of the diode due to the exponential relationship between current and electric field. Moreover, any defects present in the oxide film in the form of pinholes or traps may also give rise to undesirable conduction mechanisms such as Schottky and Poole Frenkel (PF) emission or trap assisted tunneling (TAT). Hence, growing or depositing a uniform, thin and defect-free insulator is a crucial step for efficient and reliable operation of a diode. Among several deposition methods, atomic layer deposition offers the best quality oxides with low defect density, excellent stoichiometry and superb uniformity [56]. The nano-scale patterning, to facilitate small diode area, requires the use of the most advanced ultra-fine mask-less lithographic techniques such as electron beam lithography. The latter can allow small capacitance while keeping resistance in the order of up to 100 Ω to match the antenna.

Another important point is that it is preferable to have a self-biased rectenna that operates without any external bias, so-called *zero-bias rectenna*. Furthermore, in practice, arrays of rectennas will be required to increase the collected power to useful levels. This can lead to a more efficient, higher, DC output power to the load.

Efficient IR frequency rectification requires nonlinear DC current−voltage (*I-V*) characteristics. The key MIM diode rectification figures of merit that can be determined from *I-V* characteristic are asymmetry, responsivity, nonlinearity and dynamic resistance. The asymmetry (*η_asym_*) is defined as the absolute ratio of positive ($I^{+}$) to negative current ($I^{-}$), or vice versa at a specific bias voltage:

(4) $$\eta_{asym}=\frac{\left|I^{+}\right|}{\left|I^{-}\right|}or\;\frac{\left|I^{-}\right|}{\left|I^{+}\right|}$$

Small signal rectification, however, is governed by nonlinearity around the operating point and is usually realized by square law rectification. A measure of small signal nonlinearity is responsivity, defined as the ratio of DC rectified current, *I_DC_* to input AC power, *P_in_* [57], that is

(5) $$\beta =\frac{I_{DC}}{P_{in}}=\frac{1}{2}\;{\frac{I^{\prime \prime }}{I^{\prime }}\;|}_{Vp}=\frac{1}{2}\frac{dg_{d}/dV}{g_{d}}$$

where *I′* and *I″* are the first and second derivatives of current and *g_d_* is dynamic conductance at operating point *V_p_*. Maraghechi et al. [58] have defined a nonlinearity factor as the ratio of dynamic to static conductance, that is

(6) $$\chi =\frac{dI/dV}{I/V}$$

and also used the rate of change in nonlinearity to reflect the small signal nonlinearity. The dynamic resistance is defined as the inverse of the derivative of the current with respect to the applied voltage:

(7) $$R_{D}={\left(\frac{dI}{dV}\right)}^{-1}\;$$

For MIM diodes, a particular interest is in dynamic resistance (*R*_0_ = 1/*I*′) and responsivity (*β*_0_ = *I*″/(2*I*′)) near zero-bias, as the self-biasing voltage is around millivolt. The latter is small due to generally poor coupling efficiencies that reduce the input power delivered to the MIM diode. Optimizing the parameters defined by Equations (4)–(7) from the DC *I-V* characteristics of the MIM diode can help in an improvement in the rectenna device performance under IR illumination.

This paper will present an overview of DC rectification parameters in the state-of-the-art single, double and triple MIM diodes with the aim of providing an outlook on their feasibility in IR nano-rectennas for real-life applications.

## 2. Overview of Metal Insulator Metal Diodes as Terahertz Rectifiers

### 2.1. Single Insulator MIM Diodes

**Figure 4:**
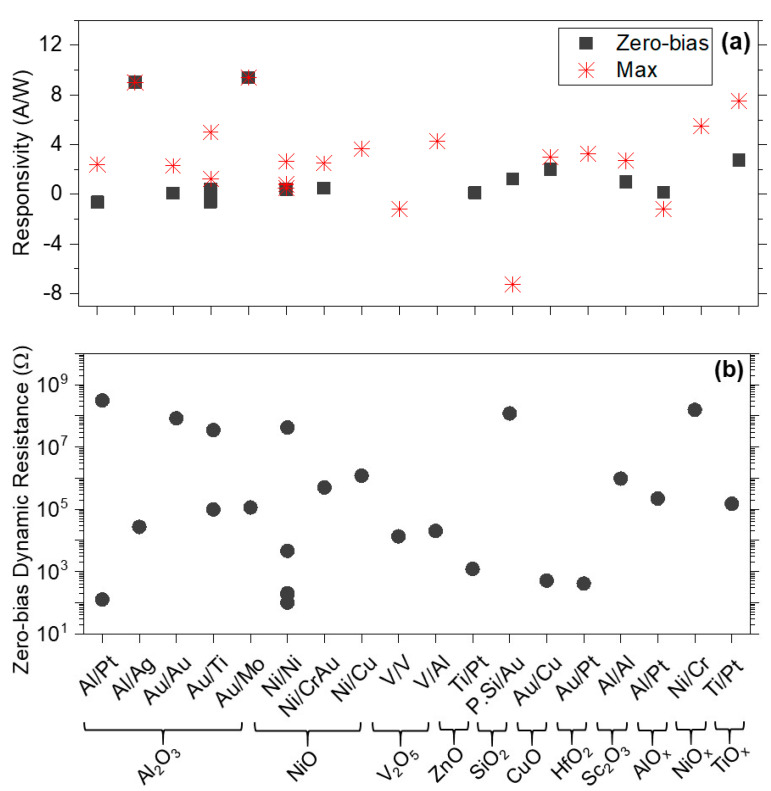
(**a**) Responsivity and (**b**) dynamic resistance for various MIM diodes. Full details are summarized in Table 2.

Various MIM diodes with different oxide layers and metal electrodes have been fabricated and characterized as depicted in Figure 4. A range of values for rectification figures of merit have been reported depending on the selection of materials, thickness of oxides, size of diodes and fabrication techniques, as listed in Table 2. It can be seen from Figure 4 and Table 2 that NiO [27,28,59,60,61,62,63,64,65,66,67,68,69] and Al_2_O_3_ [9,65,70,71,72,73,74,75] have been explored the most. The early work of Wilke et al. [27] and Fumeaux et al. [28] demonstrated the fabrication of ultra-small area diodes of 0.056 and 0.012 µm^2^, respectively, based on ~3.5 nm NiO combined with dipole, bowtie and spiral antennas. Although they demonstrated the operation of thin-film diode as mixers of 28 THz radiation for the first-time, there were issues with yield and repeatability of the fabrication process as well as low responsivity.

Hobbs et al. [76] demonstrated better responsivity and improved quantum efficiencies of 6% of waveguide-integrated near-infrared detectors based on antennas made of a multilayer Ni/Au stack that combines good IR properties of Au with the very low tunnel barrier (0.2 eV) of the Ni/NiO in MIM diodes. The geometric field enhancement technique in a Ni/NiO/Ni has been used by Choi et al. [61] to lower tunneling resistance and enhance the effective AC signal amplitude; The responsivity for this diode was superior to previously reported. Using Ni antennas makes it easy to grow NiO; However, Ni is very lossy in the infrared, especially at shorter wavelengths; Hence, there has been a resurgence of interest in fabricating NiO based diodes with other metals, such as Ag [64], Pt [65], CrAu [66,67], Cu [68] and Mo [69]. Krishnan et al. [66] realized a highly-sensitive diode (1.45 µm^2^ contact area) showing *β_MAX_* = 2.5 A/W at 0.1 V and zero-bias resistance of 500 kΩ using Ni/CrAu electrodes. By combining Ni/Cu electrochemical deposition and thermal oxidation for 2–12 nm NiO, Zhang et al. [68] reported diodes with a small area of 0.008 µm^2^, maximum responsivity of 3.65 A/W at 0.1 V but very high *R*_0_ of 1.2 MΩ. The responsivity could be further increased to 4.25 A/W by utilizing the same deposition technique for 6 nm NiO but using Ni/Ag [64]. Kaur et al. [69] reported reduced dynamic resistance to 6 kΩ when Ni/Mo electrodes were used and plasma oxidation for thin NiO film on a flexible substrate. Very high sensitivity (*S =* 2 *×* *β*) of 35 V^−1^ and resistivity of ~100 Ω at 0.6 V have been reported for Ni/NiO/Au diodes fabricated by the Langmuir–Blodgett method [63]; However, no zero-bias values are stated.

Alumina (Al_2_O_3_) is another oxide contender for MIM high-frequency applications [9,65,70,71,72,73,74,75]. Kinzel et al. [72] have demonstrated a slot-antenna-based frequency selective surface with integrated Al/Al_2_O_3_/Pt diodes showing zero-bias resistivity of 124.6 Ω. Bean et al. [71] have fabricated a dipole antenna-coupled Al/Al_2_O_3_/Pt detector using electron beam lithography and shadow evaporation metal deposition. Its specific detectivity for 28.3 THz radiation of 2.15 × 10^6^ cmHz^1/2^W^−1^ has been found to exceed IR detector performance based on Ni/NiO/Ni with 1 × 10^6^ cmHz^1/2^W^−1^ [59]. The highest zero-bias responsivity of ~9 A/W for Al_2_O_3_-based MIMs was achieved with Al/Ag [73] and Au/Mo [74] metal electrodes. Jayaswal et al. [9] designed a 28.3 THz rectenna using a bowtie nano-antenna coupled with the Au/Al_2_O_3_/Ti diode. Its zero-bias responsivity of 0.44 A/W and dynamic resistance of ~98 kΩ yielded an overall efficiency of the rectenna of 2.05 × 10^−14^.

There are a few studies of non-stoichiometric AlO_x_ [29,77], TiO_x_ [78,79] and NiO_x_ [80] based MIMs, where the fabrication parameters are varied to control oxide thickness and hence optimize device responsivity and resistance. A very high asymmetry of 2500 and low zero-bias resistivity of 600 Ω have been achieved with Al/AlO_x_/Gr (graphene) electrodes [29]. The dipole antenna-coupled Al/AlO_x_/Pt has been demonstrated with associated IR detectivity of 9.65 × 10^6^ cmHz^1/2^W^−1^ [77].

Other oxides that have been considered for inclusion in MIM diodes include ZnO [81,82], V_2_O_5_ [20,83], SiO_2_ [84,85], Nb_2_O_5_ [86,87], CuO [8], TiO_2_ [69], Cr_2_O_3_ [88], HfO_2_ [89] and Sc_2_O_3_ [90]. A simple process for fabricating planar-type MIM tunneling diodes using electron beam writing and a boiling water oxidation process has been proposed, achieving high diode sensitivity of −31 V^−1^ for Poly Si/PolySi [85] and −14.5 V^−1^ for PolySi/Au electrodes [84] but too high *R*_0_. Very high asymmetry of 7700 at 0.5 V [87] and 9000 at 1 V [79] have been reported for Nb/Nb_2_O_5_/Pt and Gr/TiO_x_/Ti, respectively; however, no *β*_0_ and *R*_0_ were reported for these diodes. Gadalla et al. [8] demonstrated an Au/0.7 nm CuO/Cu diode with *β*_0_ = 2 A/W and *R*_0_ = 500 Ω. A similar low *R*_0_ of 405 Ω has been achieved by using Au/6 nm HfO_2_/Pt diode [89].

In summary, some diodes show high responsivity but also high dynamic resistance, which is undesirable for rectifying IR energy. An alternative way to enhance the figures of merit of MIM diodes is by using multiple insulators, which is now further discussed.

### 2.2. Multiple Insulator MI^n^M Diodes, n = 2 and 3

**Figure 5:**
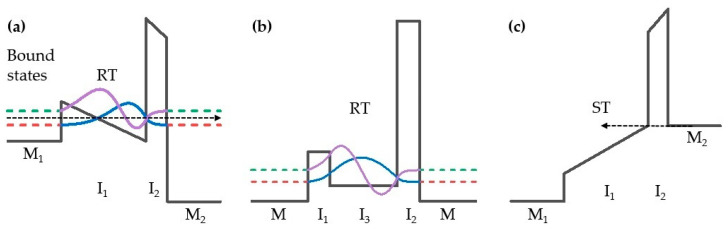
Schematics of band alignment for: (**a**) MI^2^M diode under positive bias on metal 2 showing bound states in a quantum well and conditions for resonant tunneling to occur; (**b**) MI^3^M diode under zero bias, depicting existence of bound states in a deep quantum well; (**c**) MI^2^M diode under negative bias on metal 2 and conditions of step tunneling.

The performance of MIM diodes can be enhanced by using multiple insulator diodes (MI^n^M) [91] that increase the nonlinearity of the *I-V* characteristics. There are two mechanisms that allow MI^n^M diodes to have a high responsivity while keeping the resistance low [51,56,75,91]. First is to exploit the use of resonant tunneling (RT) of electrons through a quantum well formed between the two or three insulators (Figure 5a,b). In MI^2^M, this occurs when the metal Fermi level on the higher barrier side is positively biased, creating a right-triangular well at the interface of the two insulators (Figure 5a). Moreover, Figure 5b depicts a non-cascaded triple-insulator diode, where the deep quantum well already exists even at zero bias. There are localized eigenstates in this quantum well that are referred to as bound states and electrons can propagate through these energy states enhancing current transport [92,93].

On the other hand, step tunneling (ST) occurs for the opposite bias polarity in MI^2^M shown in Figure 5c, where an abrupt increase in current occurs when the metal Fermi level on the higher barrier side rises above the conduction band of the lower barrier, thereby decreasing the tunnel distance. In a particular device, the choice of insulator materials, metals and thicknesses determines the mechanism that dominates [18,51,56,58,94,95].

**Figure 6:**
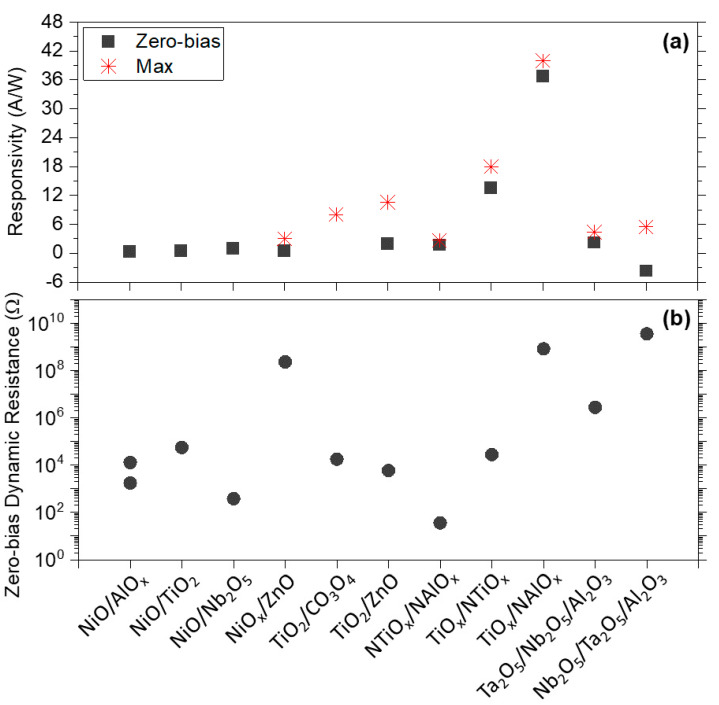
(**a**) Responsivity and (**b**) zero-bias dynamic resistance for MI^n^M diodes. For full details, please see Table 3.

Figure 6 shows responsivity and zero-bias dynamic resistance values for MI^n^M diodes (n = 2, 3) where both parameters have been reported.

Full details of rectification parameters, area and deposition technique for MI^n^M diodes are listed in Table 3 for completeness. It can be seen that one of the MIM oxide contenders, Al_2_O_3_, has been explored in combination with lower band gap oxides, such as HfO_2_ [18,58], Ta_2_O_5_ [30,51,96], Nb_2_O_5_ [17,51], and most recently NiO [45,97]. Furthermore, NiO has been used in combination with TiO_2_ [14], Nb_2_O_5_ [98] and ZnO [80]. Recent work also explores MI^2^M diodes with TiO_2_ in combination with ZnO [99], TiO_x_ [100] and Co_3_O_4_ [15], as well as nitrogen-doped TiO_2_ and Al_2_O_3_ films in a Pt/NTiO_x_/NAlO_x_/Al device [101].

The enhanced rectifying performance of a double insulator in comparison to single insulator diodes has been reported for Cr/Al_2_O_3_/HfO_2_/Cr diode by Maraghechi et al. [58]. Although promising in terms of enhanced asymmetry (>10 at 3 V), the nonlinearity at low bias was not engineered. Alimardani et al. [18,30] took a step forward in demonstrating experimentally the step tunneling mechanism in MI^2^M diodes based on Al_2_O_3_/HfO_2_ and Al_2_O_3_/Ta_2_O_5_ with a large work function difference (~0.6 eV) of metal electrodes, Al and ZCAN. Improved asymmetry and nonlinearity values were obtained at lower bias voltages (10 at 0.45 V); however, no zero-bias rectification parameters were reported. High asymmetry values of 18 at 0.35 V [51] and maximum responsivity of 6 A/W have been reported for Al/Al_2_O_3_/Ta_2_O_5_/Al, where a sharp increase in current at ~2 V has been ascribed to resonant tunneling. The latter was also observed in a Ni/NiO_x_/ZnO/Cr diode [80], showing high asymmetry of 16 at 0.5 V and *β_MAX_* = 8 A/W.

Mitrovic et al. [17] have further demonstrated a superior low-bias asymmetry of 35 at 0.1 V and a responsivity of 5 A/W at 0.25 V for the Nb/4 nm Nb_2_O_5_/1nm Al_2_O_3_/Ag diode. Moreover, the onset of strong resonance in the sub-V regime (<1 V) was found to be controlled by a work function difference of Nb/Ag electrodes in agreement with the experimental band alignment and theoretical model [17]. The model for calculating the bound states in a quantum well has been established [92,93], based on a modified multibarrier Tsu–Esaki method, whereby the insulator stack is assumed to consist of multiple slices with different barrier heights. The transmission amplitude at each energy level is found by solving the time-independent Schrodinger equation using the transmission matrix method. Using this model, Noureddine et al. [96] have also studied the effect of resonant tunneling on asymmetric Al/Ta_2_O_5_/Al_2_O_3_/Cr diodes with varied oxide thickness ratios 1:1, 1:2, 1:3 and 1:4 (in nm). They observed a good correlation between the thickness ratio of the insulating layers and the simulated bound states between the Ta_2_O_5_/Al_2_O_3_ conduction bands. The rectifying characteristics of the diodes have been improved at low turn-on voltages down to 0.17 V [102]. It is worth mentioning that none of the devices reported above were of adequately small area, which is required for integration with the antenna part; rather different metal/oxide configurations were used to engineer a diode with improved asymmetry and nonlinearity.

Herner et al. [15] investigated the relationship between responsivity and resistance in MI^2^M diodes. They fabricated Co/Co_3_O_4_/TiO_2_/Ti diodes of various thicknesses and under different annealing temperatures. A significant reduction in the dynamic resistance with a slight decrease in the responsivity has been observed after annealing of the samples up to 256 °C in air. The best performing diodes have *β*_0_ = 2.2 A/W and *R*_0_ = 18 kΩ (Table 3). In a later study [14], the zero-bias rectification performance of a Co_3_O_4_-based diode was compared to a Ni/NiO/TiO_2_/Cr structure. A theoretical quantum mechanical MIM diode simulator was used to analyze the responsivity-resistance correlation for both diodes by varying the insulator thickness. Step tunneling has been observed as the dominant conduction mechanism in both structures rather than resonant tunneling. It has been concluded that resonant tunneling is a crucial factor in reducing the dynamic resistance. The latter could be achieved by increasing the Co_3_O_4_ thickness but comes with a requirement of a higher bias voltage. Another alternative suggested in [14] is the use of a so-called geometric diode [4], but this work is outside the scope of this paper.

Pelz et al. [98] fabricated a travelling-wave diode (TWD) composed of Ni/NiO/Nb_2_O_5_/CrAu to demonstrate that the transmission line impedance can overcome the RC time constant limitations of the conventional MIM diodes at optical frequencies. According to DC *I-V* measurements, dynamic resistance of 380 Ω and responsivity of 0.46 A/W were achieved at zero-bias (Table 3). In the optical measurements, the TWD exhibited peak responsivity of 130 µA/W and the detectivity of 1.0 × 10^4^ jones. Elsharabasy et al. [99] demonstrated a Ti/TiO_2_/ZnO/Al diode with a peak responsivity of 10.6 A/W at 0.15 V, *R*_0_ = 5.9 kΩ and *β*_0_ = 1.9 A/W. Their optimized rectenna design parameters have been determined by a genetic algorithm and found to have theoretically 5.5% coupling efficiency, 6.4 A/W responsivity and 34 THz cut-off frequency.

A recent important report by Matsuura et al. [100] demonstrates Pt/TiO_2_/TiO_1.4_/Ti asymmetric diodes composed of stoichiometric and non-stoichiometric oxide layers with the aim of increasing current density and hence asymmetry. The latter increase has been found for non-stoichiometric TiO_x_, where the diode exhibited a current density of 4.6 × 10^6^ A/m^2^ and a peak asymmetry of 7.26 at 0.45 V. By exploring a similar concept as in [100], Weerakkody et al. [45] found that Ni/NiO/AlO_x_/CrAu diodes could achieve low *R*_0_ = 1.75 kΩ and reasonable high *β*_0_ = 0.31 A/W. This was achieved by engineering the electron affinity of Al_2_O_3_ by modifying its deposition conditions so that it comprises mostly of Al^3+^ ions and hence has a higher electron affinity value of 3.26 eV (the value for thin stoichiometric Al_2_O_3_ is ~1.6 eV [17]) and hence a much lower barrier with NiO. The bowtie antenna realized with this diode, designed to operate at 28.3 THz, has shown significant improvement in overall conversion efficiency of 3.7 × 10^−8^% and detectivity of 1.7 × 10^5^ cmHz^1/2^W^−1^. Another recent breakthrough is that resonant quasi-bound states can be reached at near 0 V, where Ni/NiO/AlO_x_/CrAu diodes self-bias when illuminated at 30 THz by the antenna part. By modifying the depth and width of the quantum well (Figure 5a) of a 0.035 μm^2^ diode by changing insulator thicknesses, low *R*_0_ = 13 kΩ and high *β*_0_ = 0.5 A/W were achieved simultaneously [97]. The resulting bowtie rectenna for diodes where RT has occurred shows improved power conversion efficiency of 1.7 × 10^−8^% [97]. The calculated coupling efficiency for this rectenna is found to be 5.1%, the highest achieved to date.

Another recent approach is defect engineering in MI^2^M diodes [101]. Nitrogen doping of TiO_2_ and Al_2_O_3_ using plasma-assisted ALD (PA-ALD) causes the generation of electron traps, which can assist unidirectional, defect-mediated PF transport and TAT in a multi-insulator stack. Although the latter have been found to increase rectifying performance of the doped diodes, it should be noted that the electron transport is considerably slower than tunneling, which could limit the frequency response. The best performing Pt/NTiO_x_/NAlO_x_/Al diode exhibits *R*_0_ = 36 Ω and *β*_0_ = 1.7 A/W.

The most recent theoretical study by Elsharabasy et al. [103] shows the optimization of the responsivity of MI^2^M diodes by considering metal/oxide properties and fixing the diode resistance to 100 Ω to match the nano-antenna impedance. The optimization has been performed to ensure zero-bias operation, and the diode configuration that fits the closest to the optimal solution has been found to be Ti/1 nm TiO_2_/1 nm Nb_2_O_5_/Ti, showing *R*_0_ = 108.6 Ω and *β*_0_ = 4.99 A/W from simulations. The RC time constant was found to be 9 fs for the diode area of 0.01 μm^2^, resulting in a 17 THz cut-off frequency.

In contrast to MI^2^M, there are comparatively fewer studies reported on triple-insulator diodes [56,75,104]. Maraghechi et al. [104] investigated and reported the resonant tunneling phenomenon for the first time in Cr/Cr_2_O_3_/HfO_2_/Al_2_O/Cr and Cr/Cr_2_O_3_/Al_2_O_3_/HfO_2_/Cr diodes in cascaded and non-cascaded configurations, respectively. Further work of Mitrovic et al. [56,75] demonstrated cascaded (Al/Nb_2_O_5_/Al_2_O_3_/Ta_2_O_5_/Al) and non-cascaded (Al/Ta_2_O_5_/Nb_2_O_5_/Al_2_O_3_/Al) diode configurations based on ultra-thin oxide films (1–3 nm) of Nb_2_O_5_, Al_2_O_3_ and Ta_2_O_5_ deposited by ALD. The diodes show strong tunneling and RT behavior at low voltages (0.35 V for non-cascaded configuration), substantiating evidence of the high-quality and uniqueness of atomic layer deposition that has been used to facilitate sub-nm thickness control, low oxide defect density, excellent stoichiometry and superb uniformity. The diodes exhibited a superior low-voltage responsivity of 5 A/W at 0.2 V and asymmetry of 12 at 0.1 V as the best performing MI^3^M diodes to date. The scaling of the contact area for these diodes is underway.

## 3. Permittivity and Scaling Issues

The quality of an oxide film determines the type and magnitude of the diode current. The majority of the fabricated MIM diodes shown in Table 2 have oxide thicknesses below 5 nm, which serves to facilitate quantum-mechanical tunneling. An uneven, non-uniform insulating layer can result in current crowding and hence variability and lack of reproducibility for MIM diodes. Other conduction mechanisms, such as PF emission or TAT, may arise due to oxide defects. Hence, the formation of a uniform, ultrathin (<10 nm) and defect-free insulator layer is essential. Several oxide deposition techniques are apparent, as listed in Table 2 and Table 3: native [65], plasma [15,60,61,62,66,67,69,73,78], thermal [29,64,68], boiling water [84,85], O_2_ exposure [71,72,77] and anodic [86,87] oxidation. Here, the quality of the grown oxide depends on the surface roughness of the bottom metal electrode and largely on the method of the oxide layer realization. Native oxidation is the easiest but generally yields poor-quality, non-uniform oxides due to varying conditions of humidity and partial pressure of oxygen in the air. During thermal oxidation, the bottom metal electrodes are exposed to elevated temperature to form their oxides. Such layers are also prone to the formation of pin-holes due to surface contamination. Plasma oxidation is more reliable and reproducible due to the ability to control process parameters, such as rate of oxygen flow, power and oxidation time. Anodic oxidation or anodization has also been shown to produce high-quality oxidized metal surfaces with good control of thickness [86,87]. The constraint of only growing a derivative oxide layer of the underlying polycrystalline metal can be resolved by directly depositing an insulator on the bottom metal electrode using different deposition techniques and thus facilitating the use of any type of bottom metal electrode irrespective of its native oxide formation properties. These deposition techniques include sputtering [14,17,20,27,28,45,51,59,74,80,83,90,97,98], electron beam evaporation [88] and atomic layer deposition [8,9,18,30,51,56,58,75,81,89,96,99,100,101], as listed in Table 2 and Table 3. Among these techniques, ALD offers the best quality oxides with low defect density, excellent conformality and uniformity. The ALD process involves different reactive gases used as precursors to deposit the target material. It is a self-saturating process where the insulator is grown one atomic layer at a time, providing a very precise control over thickness. Hence, it facilitates well-controlled stoichiometry and repeatability. These features have made ALD the most compatible insulator deposition technique in MIM fabrication. In addition, some other techniques have also been investigated for oxide deposition in MIM diodes, such as Langmuir–Blodgett [63,82]. Although this method facilitates easy and low-cost oxide deposition with appropriate thickness control, it has been mostly used to fabricate organic material-based insulator films [29].

Different deposition methods with associated process conditions result in variations of film homogeneity, degree of amorphousness, roughness and stoichiometry. Hence, the measured band gap and permittivity can vary even for similarly prepared oxide films. Table 4 depicts measured values of static and dynamic permittivity for most commonly used oxides in MIM diodes for rectenna, such as NiO [105,106,107,108,109], Al_2_O_3_ [110,111,112,113,114,115,116,117,118,119], ZnO [108,120,121,122], TiO_2_ [114,119,123,124,125,126,127,128], CuO [8,129,130], Ta_2_O_5_ [91,108,113,126,131], Nb_2_O_5_ [17,51,91,108], Cr_2_O_3_ [132,133], SiO_2_ [114,119], HfO_2_ [114,134,135], V_2_O_5_ [136] and Sc_2_O_3_ [137,138,139].

To further examine the merit of these oxides for rectifying THz signals, the dynamic permittivity should be used to model the power transfer efficiency of an AC voltage source connected to a MIM diode. The available data for NiO, Al_2_O_3_, ZnO, TiO_2_, CuO, Ta_2_O_5_, Nb_2_O_5_ and SiO_2_ from terahertz time-domain spectroscopy [108,117,118,128], spectroscopic ellipsometry [45,119,131] reflectance [122] and transmission measurements [130] are listed in Table 4. It can be seen from Table 4 that some of the oxides with extremely high static permittivity, such as CuO, exhibit very small dynamic permittivity justifying its use in rectenna devices [8]. Another observation is that the permittivity can depend on the thickness of the insulator, for example, it has been reported that a 2 nm TiO_x_ film exhibits a permittivity of 5.1 [140] in comparison to values of 60 [123] to 100 [127] reported for thick TiO_2_ films.

Energy conversation through the diode rectifier occurs by means of the resistance difference between the forward and reverse bias currents. Thus, the received AC signal is converted to a DC voltage. The efficiency of a rectenna can be calculated as

(8) $$\eta =\eta_{a}\eta_{s}\eta_{c}\eta_{j}$$

where *η_a_* is the coupling efficiency of incident electromagnetic radiation to the receiving antenna, *η_s_* is the efficiency of collected energy in the diode-antenna junction, *η_c_* is the power coupling efficiency between the diode and antenna and *η_j_* is the diode rectifying efficiency, which is determined by device responsivity defined by Equation (5). The power coupling efficiency (*η_c_*) between the diode and the antenna at a specific angular frequency *ω* can be calculated by

(9) $$\eta_{c}=\frac{4\frac{R_{A}R_{D}}{{\left(R_{A}+R_{D}\right)}^{2}}}{1+{\left[\omega \frac{R_{A}R_{D}}{\left(R_{A}+R_{D}\right)}C_{D}\right]}^{2}}$$

where *R*_A_ and *R*_D_ are antenna and diode resistances, respectively, and *C_D_* is the diode capacitance, which is calculated by Equation (2). It is worth mentioning that the antenna reactance is assumed to be negligible compared to the diode reactance in this equation and at high frequencies (>1 THz), this effect can reduce the coupling efficiency by a factor of ≥10 [97].

**Figure 7:**
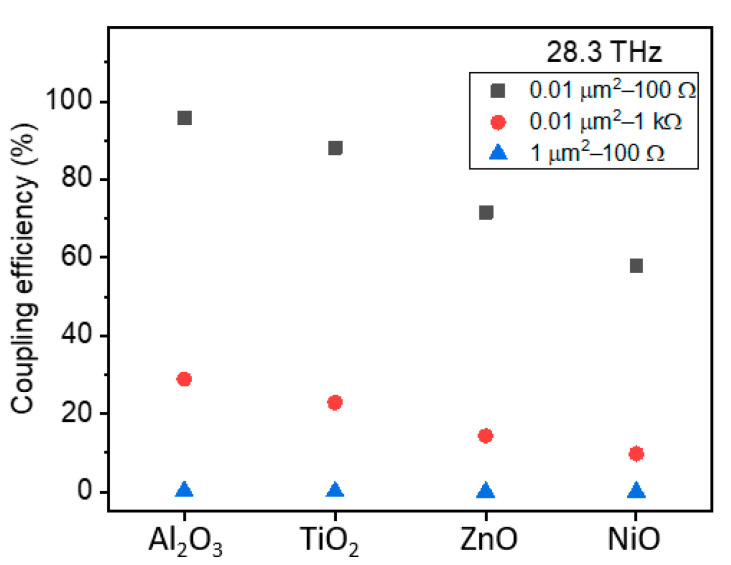
Calculated coupling efficiency for the MIM rectenna device at 28.3 THz using 3 nm oxide thickness and dynamic permittivity values as stated in Table 4.

Consider that the antenna has a resistance of 100 Ω for capturing IR radiation and the case of a full impedance match between the diode and the antenna. Furthermore, let us assume the same diode resistance as the antenna. Figure 7 depicts the power coupling efficiency at 28.3 THz of a rectenna that constitutes an MIM diode incorporating four of the most commonly used oxides.

The oxide thickness was fixed at 3 nm in the calculations. The area and the dynamic resistance values were varied between 0.01 and 1 µm^2^ and 100 Ω–1 kΩ, respectively. The high-frequency permittivity values of oxides (*ε_∞_ = n^2^*, where *n* is the refractive index) were used for the capacitance calculations [141], as stated in Table 4. As can be seen from Figure 7, the dielectrics Al_2_O_3_ and TiO_2_ come out as strong contenders with the highest coupling efficiency. An interesting observation is that the area seems to be more critical than diode resistance, that is even if the latter is engineered to be 100 Ω, the increase in area to 1 µm^2^ results in a significant reduction of coupling efficiency.

Needless to say, Figure 7 depicts an ideal case, as fabricating reliable and fully scalable MIM diodes with capacitances of a few atto-farads to operate efficiently at 28.3 THz remains a significant challenge. An emerging novel design approach is an MIM diode engineered with a junction capacitance of ~2 aF at the tip of vertically aligned multiwalled carbon nanotubes (MW-CNTs) (~10 nm in diameter), which act as the antenna [142].

By implementing an Al_2_O_3_/ZrO_2_/Al_2_O_3_/ZrO_2_ quad-insulator laminate structure, asymmetry of 300 and *β_MAX_* = 6 A/W has been achieved for the diode and a total conversion efficiency of 3 × 10^−6^% for the rectenna [143]. Although very encouraging, the growth of carbon nanotubes must be further improved to obtain a well-ordered network and to favor the amplification of the electromagnetic field structure. Variability and reproducibility are also major challenges considering that the diodes will be incorporated into large arrays to enable the generation of significant power levels.

Other emerging concepts relate to surface plasmon excitation within a MIM device that produces power based on spatial confinement of electron excitation through plasmon absorption [144]. The recent work also proposes MIM-based plasmonic structures that incorporate a nanoslit for IR rectification [145].

## 4. Conclusions and Outlook

We have presented a review of the state-of-the-art single, double and triple MIM diodes for inclusion in IR nano-rectennas. Typical metals used in MIMs (Ni, Al, Au, Cr and Ti), their skin depths and work function have been summarized. An overview of various oxides, their electron affinity, band gap and permittivity were presented as well as a review of their use in MIM diodes. The lowest zero-bias resistances have been reported for Ni/NiO/Ni (100 Ω), Al/Al_2_O_3_/Pt (125 Ω), Al/AlO_x_/Gr (600 Ω), Au/HfO_2_/Pt (405 Ω) and Au/CuO/Cu (500 Ω). Apart from the latter diode that exhibits *β*_0_ = 2 A/W, generally, these diodes have not been optimized to achieve the high zero-bias responsivity that is required for practical high-frequency operation. Hence, there has been a considerable research effort in engineering a diode with low resistance and high responsivity by utilizing resonant or step tunneling in a double or triple insulator oxide stack. The two oxide contenders for MIM diodes, NiO and Al_2_O_3_, have been utilized the most and combined with HfO_2_, Ta_2_O_5_, Nb_2_O_5_, ZnO and TiO_2_. The latter oxide has also been used in combination with Co_3_O_4_ and TiO_x_. The highest zero-bias responsivities of 2.2 and −3.7 A/W have been reported for Ti/TiO_2_/Co_3_O_4_/Co MI^2^M and Al/Nb_2_O_5_/Ta_2_O_5_/Al_2_O_3_/Al MI^3^M diodes, respectively, while the lowest zero-bias dynamic resistance of 380 Ω was reported for Ni/NiO/Nb_2_O_5_/CrAu. The latest research shows that by modifying the depth and width of the quantum well of a 0.035 μm^2^ Ni/NiO/AlO_x_/CrAu resonant tunneling MI^2^M diode, relatively low *R*_0_ = 13 kΩ and high *β*_0_ = 0.5 A/W can be achieved simultaneously. The bowtie rectenna based on this diode has been found to have overall power conversion efficiency of 1.7 × 10^−8^% and a coupling efficiency of 5.1% when illuminated at 30 THz; the highest achieved to date. Furthermore, defect engineering by nitrogen doping in a Pt/NTiO_x_/NAlO_x_/Al diode has led recently to state-of-the-art values of *R*_0_ = 36 Ω and *β*_0_ = 1.7 A/W. The most recent theoretical work indicates that a Ti/1 nm TiO_2_/1 nm Nb_2_O_5_/Ti diode could achieve even higher *β*_0_ = 4.99 A/W and low *R*_0_ = 108.6 Ω and, with the diode area of 0.01 μm^2^, could result in an efficient IR rectenna with a cut-off frequency of 17 THz.

In summary, there is no one optimal solution of metal/oxide stack combination that can yield an efficient MIM rectifier for IR rectenna due to engineering design trade-offs. Rather, several options of MI^n^M are becoming apparent. The latest research points to technological advancements and is focused on the control of oxide thicknesses to sub-nanometer accuracy and oxide stoichiometry by carefully devised fabrication processes, which have resulted in realizing some of the best zero-bias responsivity–resistance optimized diodes. Although there is considerable scope for refinement in device manufacturing, the latest research in this field shows that we are a step closer toward tapping into the infrared spectrum with rectennas based on MIM technology. The race, however, is still on.

## Figures and Tables

**Table 1:** Physical properties of metals and oxides: work function, electron affinity and band gap.

Metal	Work Function (eV)	Oxide	Electron Affinity, *χ* (eV)	Band Gap (eV)
Ni	4.9 [11,12], 4.99 [13], 5.04–5.35 [14]	NiO	1.46 [32], 3.0 [33]	3.4 [45], 3.8 [33], 4.0 [46]
Co	4.00 [11], 4.8 [13], 5.00 [15]	Al_2_O_3_	1.57 [17], 2.58 [34], 3.50 [9]	5.95 [45], 6.4 [38,47]
Ag	4.26 [16,17], 4.33 [12], 4.7 [11]	ZnO	4.3–4.5 [35], 4.6 [33]	3.2 [48], 3.38 [35], 3.4 [33]
Cu	4.5 [11,12], 4.63 [13], 4.65 [16]	TiO_2_	3.9 [36], 4.0 [33]	3.05 [36], 3.2 [33], 3.3 [49]
Au	4.8 [11], 5.1 [16], 5.28 [12]	CuO	4.07 [37]	1.2–1.8 [50]
Al	4.2 [11,18], 4.28 [16,17]	Ta_2_O_5_	3.2 [36], 3.3 [33], 3.75 [35]	4.17 [35], 4.4 [36,38], 4.45 [51]
W	4.5 [11,19], 4.8 [13]	Nb_2_O_5_	3.72 [17], 4.23 [38]	3.71 [51], 3.8 [38]
Mo	4.2 [11], 4.4 [13], 4.6 [16]	Cr_2_O_3_	3.16–4.05 [39]	3.2 [52], 3.24 [53]
Zn	4.3 [11,16]	Co_3_O_4_	3.05–4.05 [40]	2.10 [54]
Pd	5.0 [11], 5.12 [12]	SiO_2_	0.9 [33,36]	8.8 [38], 9.0 [33]
Pt	5.3 [11], 5.65 [16]	HfO_2_	2.2 [33], 2.25 [41]	5.6 [38], 6.0 [36]
Cr	4.0 [13], 4.4 [11], 4.5 [16]	V_2_O_5_	7.1 [42]	2.36 [55]
Ta	4.1 [11,13], 4.25 [16]	Sc_2_O_3_	2.06 [43], 1.98–2.5 [44]	5.7–6.0 [43]
Nb	4.1 [17], 4.3 [16]	-	-	-
V	4.44 [20]	-	-	-
Ti	3.7 [13], 4.1 [11], 4.33 [16]	-	-	-

**Table 2:** A summary of rectification parameters for state-of-the-art MIM diodes, including the device area, oxide thickness and deposition technique.

Oxides	Metals	*β*_0_ (A/W)	*β_MAX_* (A/W)	*R*_0_ (Ω)	*η_asym_*	Area (μm^2^)	Thickness (nm)	Deposition Technique
NiO	Ni/Ni [27]	-	0.8	200	-	0.056	3.3	Sputtering
	Ni/Ni [28]	-	-	100	-	0.012	~3.5	Sputtering
	Ni/Ni [59]	-	0.825	180	-	0.075	3.5	Sputtering
	Ni/Ni [60]	-	0.5	~4.6 k	-	0.01192	2.5	Plasma oxidation
	Ni/Ni [61]	−0.41	−2.65	42.4 M	-	0.018	<4	Plasma oxidation
	Ni/Au [62]	2.8	4.56	-	-	0.64	2.2	Plasma oxidation
	Ni/Au [63]	-	17.5	-	22 at 0.6 V	4.4 × 10^−5^	2.6–4.2	Langmuir-Blodgett
	Ni/Ag [64]	2.9	4.25	-	4.7 at 1.0 V	3.1 × 10^−4^	6	Thermal oxidation
	Ni/Pt [65]	−1.5	−6.5	-	-	0.075	1–2	Native oxidation
	Ni/CrAu [66]	0.5	2.5	500 k	-	1.45	~3	Plasma oxidation
	Ni/CrAu [67]	-	-	-	6 at 0.2 V	100	5.5	Plasma oxidation
	Ni/Cu [68]	-	3.65	1.2 M	-	0.008	12	Thermal oxidation
	Ni/Mo [69]	-	-	6 k	-	-	2.4	Plasma oxidation
Al_2_O_3_	Al/Al [65]	0.05	−0.7	-	-	-	1–2	Controlled oxidation
	Al/Ni [65]	0.25	0.5	-	-	-	1–2	Controlled oxidation
	Al/Ti [65]	0.3	1.0	-	-	-	1–2	Controlled oxidation
	Al/Pt [65]	0.5	0.65	-	-	-	1–2	Controlled oxidation
	Al/Pt [70]	0.5	2.25	-	-	0.0025	2–2.5	Controlled oxidation
	Al/Pt [71]	−0.64	2.4	312 M	-	0.004	1–2.5	O_2_ exposure
	Al/Pt [72]	-	-	125	-	-	1–2	O_2_ exposure
	Al/Ag [73]	9.0	9.0	27 k	1.2 at 0.6 V	1,760,000	-	Plasma oxidation
	Au/Mo [74]	9.4	9.4	113 k	-	1.0	~6	Sputtering
	Au/Au [75]	0.1	2.3	83 M	1.3 at 1.2 V	10,000	3	ALD
	Au/Ti [9]	0.44	1.25	98 k	-	0.04 *	1.5	ALD
	Au/Ti [75]	−0.6	5	35 M	1.7 at 1.5 V	10,000	3	ALD
AlO_x_	Al/Gr [29]	-	-	600	2500 at 1 V	-	~3	Thermal oxidation
	Al/Pt [77]	~0.15	−1.2	~220 k	-	0.0056 *	~2 nm	O_2_ exposure
TiO_x_	Ti/Pt [78]	2.75	7.5	~150 k	-	-	-	Plasma oxidation
	Gr/Ti [79]	-	-	-	9000 at 1 V	12	-	Thermal oxidation
NiO_x_	Ni/Cr [80]	-	5.5	157 M		400	7	Sputtering
ZnO	Ti/Pt [81]	0.125	-	1.2 k	-	90,000	4	ALD
	AuCr/Ni [82]	-	16	-	12 at 0.78 V	100	~4	Langmuir-Blodgett
V_2_O_5_	V/Al [20]	-	4.26	20 k	-	4.0	3	Sputtering
	V/V [83]	-	−1.18	13.4 k	-	4.0	1.45	Sputtering
SiO_2_	PolySi/Au [84]	~1.25	−7.25	120 M	5 at 0.4 V	0.35	1.38	Boiling water oxidation
	PolySi/PolySi [85]	~1.5	−15.5	-	-	6 × 10^−5^	-	Boiling water oxidation
Nb_2_O_5_	Nb/Pt [86]	-	10	-	1500 at 0.5 V	45,239 *	15	Anodic oxidation
	Nb/Pt [87]	-	8.45	-	7700 at 0.5 V	6400 *	15	Anodic oxidation
CuO	Au/Cu [8]	2.0	3.0	500	-	0.004489	0.7	ALD
TiO_2_	Ti/Pd [69]	-	-	100 k	-	-	3	Plasma oxidation
Cr_2_O_3_	Au/Cr [88]	-	4.0	-	-	-	5	Electron beam evaporation
HfO_2_	Au/Pt [89]	-	3.29	405	-	4.0	6	ALD
Sc_2_O_3_	Al/Al [90]	1.0	2.7	960 k	1.3 at 1.2 V	10,000	3	Sputtering

* Device area calculated based on stated dimensions.

**Table 3:** A summary of device and rectification parameters for multiple insulator MI^n^M diodes, n = 1, 2 including device area, oxide thickness in the insulator stack and oxide deposition technique.

Oxides	Metals	*β*_0_ (A/W)	*β_MAX_* (A/W)	*R*_0_ (Ω)	*η_asym_*	Area (µm^2^)	Thickness (nm)	Deposition Technique
Al_2_O_3_/HfO_2_	ZCAN/Al [18]	-	-	-	>10 at 0.8 V	8 × 10^5^	2.5/1	ALD
	Cr/Cr [58]	-	~2.5	-	~10 at 3 V	2 × 10^5^ *	2/2	ALD
Al_2_O_3_/Ta_2_O_5_	ZCAN/Al [30]	-	-	-	10 at 0.45 V 187 at 1.2 V	8 × 10^5^	2.5/2.5	ALD
	Al/Al [51]	-	6.0	-	18 at 0.35 V	1 × 10^4^	1/4	Sputtering/ALD
	Cr/Al [96]	-	-	>10^7^ M	~8 at ~1 V	1 × 10^4^	1/4	ALD
Al_2_O_3_/Nb_2_O_5_	Ag/Nb [17]	-	8.0	-	35.2 at 0.06 V	1 × 10^4^	1/4	Sputtering
	Al/Al [51]	-	9.0	-	7.6 at ~0 V	1 × 10^4^	1/4	Sputtering/ALD
NiO/AlO_x_	Ni/CrAu [45]	0.31	-	1.75 k	-	0.025	2/1.1	Sputtering
	Ni/CrAu [97]	0.5	-	13 k	-	0.035	4/1	Sputtering
NiO/TiO_2_	Ni/Cr [14]	~1.0	-	56 k	-	0.071	-	Sputtering/ O_2_ ambient
NiO/Nb_2_O_5_	Ni/CrAu [98]	0.46	~3.0	380	~1.15 at 0.2 V	0.1552 *	3/2	Sputtering/ O_2_ ambient
NiO_x_/ZnO	Ni/Cr [80]	-	8.0	234 M	16 at 0.5 V	400 *	7/7	Sputtering
ZnO/TiO_2_	Al/Ti [99]	1.9	10.6	5.9 k	-	72.27 *	0.5/1.65	ALD
TiO_2_/Co_3_O_4_	Ti/Co [15]	2.2	4.4	18 k	1.2 at 0.1 V	0.071	2.7/2.5	Plasma oxidation/sputtering
TiO_2_/TiO_1.4_	Pt/Ti [100]	-	-	-	7.26 at 0.45 V	900	3/2	Annealing/ALD
NTiO_x_/NAlO_x_	Pt/Al [101]	1.7	2.7	36	1 at 0.5 V	100	7/3	PA-ALD
Ta_2_O_5_/Nb_2_O_5_/Al_2_O_3_	Al/Al [56]	-	5.1	-	12 at 0.1 V	1 × 10^4^	2/2/1	ALD
Ta_2_O_5_/Nb_2_O_5_/Al_2_O_3_	Al/Al [75]	1.2	4.3	2.8 M	4.3 at 1.6 V	1 × 10^4^	1/3/1	ALD
Nb_2_O_5_/Ta_2_O_5_/Al_2_O_3_	Al/Al [75]	−3.7	5.5	3.6 G	117 at 1.6 V 6 at 0.1 V	1 × 10^4^	1/3/1	ALD

* Device area calculated based on stated dimensions.

**Table 4:** Static and dynamic permittivity (at ~1 THz and 28.3 THz) of most common oxides used in MIM diodes for rectennas.

Oxide	Permittivity (*ε_r_*)	Dynamic Permittivity (*ε_∞_*)	
		1–3 THz	28.3 THz
NiO	7.9–16.7 [105], 11.9 [106,107]	9.6 [108]	3.24 [109]
Al_2_O_3_	7 [110], 7.6 [111], 8.3 [112], 8.9 [113], 9 [114], 10 [17,51,115,116]	~9 [117], 11.5 [118]	0.8 [45,119]
ZnO	8.5 [120], 9.4–10.4 [121]	7.0 [108]	2.4 [122]
TiO_2_	60 [123], 70 [124], 80 [114,125], 88–102 [126], 100 [127]	~100 [128]	1.4 [119]
CuO	10^3^–10^5^ [129]	-	2.4 [8,130]
Ta_2_O_5_	20 [91], 23.9 [113], 25 [126]	~33 [108], 22.7 [131]	-
Nb_2_O_5_	25 [17,51,91]	~22 [108]	-
Cr_2_O_3_	10.3 [132], 11.8–13.3 [133]	-	-
SiO_2_	3.9 [114]	-	4.7 [119]
HfO_2_	14 [134], 18 [135], 25 [114]	-	-
V_2_O_5_	11.5–22.3 [136]		
Sc_2_O_3_	8.5–9.3 [137], 14 [138,139]		

## Data Availability

Not applicable.
